# Concomitant procedure for coronary artery disease and aorto iliac vessel block with an non - healing ulcer over the foot

**DOI:** 10.1186/1749-8090-10-S1-A58

**Published:** 2015-12-16

**Authors:** Durgaprasad Reddy, SR Vikas, S Mayuri, Subhash Kumar

**Affiliations:** 1 Department of Cardiothoracic and Vascular Surgery, Vydehi Institute of Medical Sciences and Research Centre, Bangalore, Karnataka, 560066, India

## Background/Introduction

The prevalence of Coronary artery disease (CAD) in patients with peripheral vascular disease (PVD) varies widely from 28% to 94% in published reports.

## Aims/Objectives

We share our experience with a concomitant procedure for coronary artery disease and peripheral vascular disease using an artificial conduit from ascending aorta to peripheral vessel in a single sitting.

## Method

Records of 41 patients who underwent cardiac and peripheral vessel revascularisation between January 2009 and January 2014 were retrospectively analysed. All patients had diseased abdominal aorta with claudication pain and non-healing ulcer over the foot and a coronary angiogram showing either a triple vessel disease (27) or a double vessel disease (14). All patients underwent coronary artery bypass grafting and aorto-bifemoral grafting in a single sitting.

## Results

Post-operative Doppler study showed good peripheral blood flow in all patients. Patients were relieved from rest pain and non-healing ulcers were converted to healing ulcers and limb salvage was possible in all cases. Four patients had pericardial effusion due to weeping of graft, which was drained with the help of pig tail catheter. Two patients had serous collection at the inguinal site which required drainage. These complications did not compromise the hemodynamics of the patient in the form of cardiac tamponade or limb ischemia.

## Discussion/Conclusion

Single sitting for CAD and PVD revascularization has reduced morbidity, is easy to perform, cost effective and reduced hospital stay.

